# Association of Long-Term Pesticide Exposure and Biologic Parameters in Female Farm Workers in Tanzania: A Cross Sectional Study

**DOI:** 10.3390/toxics4040025

**Published:** 2016-09-29

**Authors:** Wilbert Bunini Manyilizu, Robinson Hammerton Mdegela, Rudovick Kazwala, Hezron Nonga, Mette Muller, Elisabeth Lie, Eystein Skjerve, Jan Ludvig Lyche

**Affiliations:** 1 Department of Veterinary Medicine and Public Health, Sokoine University of Agriculture, P. O. Box 3021, Morogoro 023, Tanzania; rmdegela2012@gmail.com (R.H.M.); kazwala@gmail.com (R.K.); nongahezron@yahoo.co.uk (H.N.); 2 Department of Food Safety and Infection Biology, Norwegian University of Life Sciences, P. O. Box 8146, Oslo 454, Norway; mette.helen.bjorge.muller@nmbu.no (M.M.); elisabeth.lie@niva.no (E.L.); eystein.skjerve@nmbu.no (E.S.); jan.l.lyche@nmbu.no (J.L.L.)

**Keywords:** pesticides, hematological, biochemical, acetylcholine esterase activity, effects

## Abstract

The study aimed to assess the association of long-term pesticide exposure (≥5 years) with hematological, serum biochemical parameters and acetylcholinesterase activity in farm workers. These pesticides included organophosphorus pesticides, carbamates, pyrethroids, dithiocarbamates, and other pesticides such as endosulfan. Applying a cross-sectional study design, 69 females from a pesticide-exposed farm population and 30 females from a district not using pesticides (reference group) were studied. The mean red cell corpuscular volume and hematocrit values were significantly lower (74.7 ± 9.1 fl; 95% CI 72.5–76.9 and 32.0% ± 4.6%; 95% CI 30.9–33.1, respectively) in the exposed compared to the reference group, whereas mean corpuscular hemoglobin concentration and platelets were significantly higher (37.4 ± 3.8 g/dL; 95% CI 36.5–38.3 and 374.1 ± 95.3/L; 95% CI 351.2–396.9, respectively) in the exposed compared to the reference group. Mean serum glutamic oxaloacetate transaminase (20.7 ± 8.9 U/L; 95% CI 18.5–22.9) and creatinine (83.9 ± 6.6 μmol/L; 95% CI 82.3–85.5) were significantly higher in the exposed compared to the reference group. A higher mean esterase activity (AChE 0.6 ± 0.2 mM/min/mg protein; 95% CI 0.56–0.7; BChE 0.9 ± 0.4 mM/min/mg protein; 95% CI 0.9–1.1) was noted in the exposed group. Regression models suggest that occupational exposure (*p* < 0.001) could be a predictor of esterase (AChE and BChE) activity and biochemical changes (β = 0.4, 95% CI: 0.3–0.5; β = 0.7, 95% CI: 0.6–0.9, respectively). Long-term pesticide exposure affects the hemato-biochemical and esterase responses, establishing the need for further studies.

## 1. Introduction

Agriculture is the economic backbone of Tanzania, providing 80% of the country’s employment and contributing to approximately 26% of the National Gross Domestic Product [1]. The use of pesticides in agricultural production is necessary in order to increase crop yield and to minimize post-harvest losses. A substantial increase in pesticide use has been documented since the 1990s [2]. A report about trade and utilization of pesticides in Tanzania [3] showed that between 2000 and 2003, the import of pesticides increased from 500 to 2500 tons; by 2006, 682 different types of pesticides were registered, including endosulfan and dieldrin, which are restricted by the Stockholm Convention due to their environmental persistence and toxic potential [4].

Use of pesticides has led to an increase in human exposure, which has been associated with acute morbidities [4,5] and mortalities. This is particularly true in developing countries [5,6], where there are poor regulations, low enforcement, difficulties in accessing information, and a lack of surveillance systems and training. Reports from African countries have shown that farm workers use pesticides with low awareness of safe handling, utilization, and protection [7]. A high degree of misuse has been documented, including over- and under-dosing, mixing of different pesticides, dangerous storage of pesticides, poor spraying equipment, and poor use of personal protective gear [8]. Such misuse of pesticides has been associated with adverse health effects among farm workers [9,10,11,12]. Most studies report acute health effects on highly exposed groups such as sprayers [9]; however, available data on potential adverse effects of long-term and frequent exposure to multiple pesticides are limited [10,11,12,13].

Exposure to pesticides has been associated with central nervous, reproductive, and immune system disorders and cancers [14,15,16,17,18,19,20,21,22,23,24,25,26,27,28,29]. Since modern pesticides are rapidly cleared from the body, it is necessary to use relevant biomarkers to indicate exposure and effects.

Acetylcholinesterase (AChE) is a useful biomarker as it provides an integrated measurement of the overall neurotoxic risk of bioavailable contaminants including other environmental and occupational chemicals than organophosphates (OPs) and carbamates [30]. For example, it is reported that AChE can be affected by pyrethroid and triazine pesticides and also heavy metals like mercury, lead, cadmium, and copper. Despite several limitations, such as wide inter-individual and intra-individual variation [31], the AChE test is a useful biomarker in assessing both exposure and effects of OPs and carbamates [32]. Also, its inhibition is more sensitive than butyrylcholinesterase (BChE) in long-term exposure to OPs due to the lower recovery rate compared to BChE. This follows a cumulative inhibitory effect on AChE activity [30,33]. However, for pesticides, which do not affect AChE activity, there are no specific biomarkers of effect established.

Routine blood tests are part of a full clinical examination and are interpreted in conjunction with clinical symptoms for diagnosis of disease. Previous studies have reported associations between long-term, low-level exposure to pesticides and changes in biochemical and hematological parameters [32,34,35,36,37,38]. In these studies, exposed farm workers, including sprayers, were found to have lower mean hemoglobin levels, hematocrit concentrations and mean cell volumes, as well as higher mean corpuscular hemoglobin concentrations (MCHC) and platelet volumes. In addition, liver enzymes such as alkaline phosphatase, serum alanine aminotransferase, and aspartate aminotransferase were elevated in the exposed individuals compared to controls. However, esterase activities were inhibited. These parameters may be used as early biomarkers for adverse effects. 

In the present study, the commonly used pesticides included organophosphorus pesticides, carbamates, pyrethroids, and dithiocarbamates. Indiscriminate use and handling of pesticides and symptomatic effects of acute exposure among male farm workers have been reported; however, data on long-term exposure to mixtures of pesticides and adverse effects among females in developing countries is lacking [9,10,11,12]. However, the present study focuses on female farm workers of reproductive age who were engaged in farming activities such as weeding and harvesting, as they are also at risk of adverse health effects. The findings from the present study add to the body of knowledge for populations exposed to low doses of pesticides through various exposure pathways.

The specific objective of this study was to describe and establish the associations between long-term, frequent exposure to pesticides and potential adverse health effects in female farm workers using hematological and serum biochemical parameters and esterase activity as biomarkers of effect. We hypothesized that long-term, frequent exposure to pesticides alters biological responses including hematological and serum biochemical parameters and esterase activity in exposed individuals.

## 2. Materials and Methods

### 2.1. Study Population and Design

The study was conducted in two neighboring districts of the Arusha region in the north of Tanzania between September 2012 and February 2013. The exposed group was recruited from Karatu district, which produces mainly onions for both national and international markets and where the farmers use pesticides on a daily basis for protecting crops [11,39]. Mean pesticide uses in liters, spray frequency per crop and per week were 637 and 1.3, respectively, for about 270 days a year [39]. The reference population was recruited from a neighboring district—rural Arusha—where the inhabitants are mainly cattle farmers who grow vegetables for their own consumption without using pesticides. The sample size of the study population was determined using a significance level of 0.05, a power of 80%, an expected proportion with biologic parameters alterations in the reference group assumed at 40% (versus 70% in the exposed population), and a ratio of reference to exposed group of 1:2. The calculated minimum number of subjects was 93, with 30 reference and 63 exposed individuals. The study recruited 99 healthy adult females, of whom 69 were from Karatu (exposed group) and 30 were from rural Arusha (reference group). The recruited study participants were 20 years of age or older and were HIV-negative based on clinic records (a reproductive and child health card). They were living as farm workers and had been full-time residents of the study area for at least five years. The females in the exposed group were usually engaged in weeding and harvesting, whereas males did all other activities including pesticide spraying, mixing, and loading.

The study population was defined as exposed if they had resided for at least five years in Karatu, where indiscriminate pesticide use and pesticide poisoning cases were previously reported [11]. Other inclusion criteria for the exposed group included engaging in weeding and harvesting for at least three times a week and consuming vegetables and other produce from these farms. Inclusion criteria for the reference population included working with crop production and residing in rural Arusha for at least five years. 

Non-pregnant females from the exposed and reference groups were recruited at reproductive and child health clinics at Askofu Hhando health center and Olturumet hospital in the Arusha region. Recruitment of study subjects was achieved with the assistance of nurses in the reproductive and child health clinics. A trained nurse identified eligible clients during attendance registration, based on the inclusion and exclusion criteria, and described the study to the potential clients. Females who agreed to participate in the study signed a written consent form. 

### 2.2. Questionnaire Data

A structured questionnaire was created in English and then translated into Swahili. The questionnaire was either administered by the researcher or the local clinician. Data were collected on background characteristics such as age, education level, family income per month, occupation, tobacco smoking, drug use, and alcohol consumption. Questions were also asked about years in farm work, years of living in the study area, and names of pesticides used. Questionnaire administration was followed by a complete physical examination to check for pallor to exclude anemia, pedal edema to exclude kidney and circulatory disorder, respiratory rate and pulse rate to exclude respiratory and circulatory disorder, abdominal distension and palpation to exclude abdominal masses including kidney and liver pathology. 

### 2.3. Measurement of Blood Parameters

Blood sampling and measurements were performed by specialized laboratory professionals at the service facility who had been working in the laboratory for at least five years. A sample of 5 mL of blood was drawn from each consenting study subject. From these 5 mL, 2 mL were transferred into EDTA tubes for hematological analyses. The remaining 3 mL of blood was centrifuged at 25 °C. Plasma was then apportioned into two different tubes, one for biochemical analysis and the other for esterase activity analysis. Samples for esterase enzyme activity were stored at freezing point of −196 °C and −80 °C in the field and laboratory, respectively, until analysis.

The samples for hematology were analyzed at Mt. Meru regional referral hospital hematology laboratory in Arusha using the hematological analyzer, a MS9-3H automatic full digital cell counter machine (@ 2003 Melet Schloesing Laboratories, Cergy-Pontoise, France). Samples for biochemical analysis were analyzed at the same hospital biochemistry laboratory as per CHEM 7 Erba Diagnostic Mannheim GmbH, Germany description (Quality System Certified ISO 9001 ISO 13485) for IFCC method-kinetic without pyridoxal phosphate (serum glutamic pyruvate transaminase [Sgpt], serum glutamic oxaloacetic transaminase [Sgot]), CHOD-PAP (Cholesterol) and Jaffe’s method (Creatinine). The total protein concentration of each sample was determined using the Biuret Method (procedure described by Erba diagnostic Mannheim GmbH, Mannheim, Germany) at the Sokoine University of Agriculture (SUA) physiology laboratory, and esterase activity was measured in the toxicology laboratory. The procedure behind the documented fact is described by Ellman et al. (1961). We used 0.1 M phosphate buffer (pH 8.0), 0.075 M acetylthiocholine iodide (21.67 mg/mL) substrate for AChE and S-butyrylthiocholine iodide substrate for cholinesterase variant (BChE), 0.01 M dithiobisnitrobenzoic acid (DTNB) reagent, sodium bicarbonate, and sodium phosphate (monobasic and dibasic). The enzyme activity present in the blood plasma sample was calculated as a slope from the plot equation of linear regression [40]. Enzyme activity analysis was calculated in μmol/min/mL = ∆ absorbance/minute × total volume (in mL) × 1000/13.6 DTNB absorptivity (in mM) × 1 (light path in cm). Activity data were expressed in mM/min/mg protein. 

### 2.4. Predictor and Outcome Variables

Predictor and outcome variables were predominantly continuous. Predictor variables included age in years, height and weight, years in current occupation, and years in current residence. Outcome variables encompassing hematological parameters included white blood cell count (WBC), lymphocytes, monocytes, red blood cells, mean corpuscular volume, hematocrit, mean corpuscular hemoglobin, mean corpuscular hemoglobin concentration, and hemoglobin and platelet count. Outcome variables encompassing biochemical parameters included serum gpt and got, cholesterol and creatinine. The other outcome variable was cholinesterase activity (AChE and BChE).

### 2.5. Statistical Analysis

Subjects that had missing data were removed from the dataset before analysis. Exposed and reference groups were compared across demographic and socio-economic characteristics such as age, family income per month, and years of living in the current area using the Student’s *t* test. Comparisons between exposed and reference groups for hematological and biochemical measures were done using the two-group mean comparison sample *t*-test with unequal variances.

Bivariate and multivariate analyses were used to determine whether passive but long-term pesticide exposure was associated with abnormal cholinesterase activity or hematological and biochemical parameters. Predictor variables for linear regression models included age, body weight, family income per month, years in current occupation, and years living in the current study area. Outcome variables were the selected hematological and biochemical parameters including cholinesterase activity. The final model included predictors that had *p*-values below 0.2 in the univariate regression analysis, including age, years of living in the study area, current occupation, and body weight. The regression models were tested for fit and residual patterns using standard methods.

### 2.6. Ethics Considerations

The study obtained ethics approval from the coordinating team for National Institute for Medical Research of Tanzania. The study subjects were not sick but had attended the facility for other described reasons and no incentive was given. An informed consent was signed prior to blood sample donation and questionnaire administration. The health facility medical practitioner provided education first about the study.

## 3. Results

A total of 99 female subjects were included in the study, of which 69 were in the exposed group and 30 were in the reference group. Characteristics of females in the exposed and reference groups are shown in Table 1.

Age, years of living in the study area, and years in their current occupation for females in both groups were in the ranges of 18–45, 5–40, and 5–35 years, respectively (Table 1). Females in the exposed group had lived in the study area for fewer years (16.8 ± 8.8; 95% CI 14.5–18.9) and were older than females in the reference group (23.1 ± 9.2; 95% CI 19.6–26.5). There was no significant difference of family mean income Tanzanian shillings per month between the exposed (292,173 ± 106,769; 95% CI 266,525–317,822; *p* = 0.4) and reference groups (316,666 ± 168,325). The majority of all females (87%) had at least seven years of primary education, although 13% had no formal education. Educational status was similarly low (primary level education for seven years) in both groups with more than 20% having secondary school education in exposed females (data not shown). In addition, few women (3%) smoked and/or drank alcohol. Use of medications was not significantly different between the two groups.

The commonly applied pesticides in the study area are summarized in Table 2. 

Table 3 and Table 4 show the distribution of hematological and biochemical parameters and esterase activity in the exposed and reference groups. White blood cell, lymphocyte, and platelet counts were significantly higher (6.8 ± 2.3, 2.7 ± 0.8, 374.1 ± 95.3) in the exposed group than in the reference group (5.3 ± 1.2, 2.2 ± 0.4, 259.6 ± 77.4), although all counts were within normal ranges. Mean corpuscular volume and hematocrit were significantly lower in the exposed group (74.7 ± 9.1, 32.0 ± 4.6) compared to the reference group (84.7 ± 7.6, 36.2 ± 4.8; *p* < 0.05). The exposed group had higher mean levels of lymphocytes, monocytes, platelets and serum glutamic oxaloacetic acid transaminase, and serum creatinine than the reference group. Females in the exposed group had higher AChE and BChE activity than females in the reference group (*p* < 0.01 for both measures). More specifically, the BChE activity in the exposed group was four times higher than in the reference group.

Multivariable regression models adjusted for age and years of living showed that females in the exposed group had significantly higher levels of both AChE and BChE activities (β = 0.4, 95% CI: 0.3–0.5; β = 0.7, 95% CI: 0.6–0.9, respectively) and non-significant differences for Sgot and cholesterol (β = 2.0, 95% CI: 5.2–9.1; β = 0.4, 95% CI: 0.1–0.9, respectively) compared to females in the reference group. After adjusting the models for age and years of living in the study area, exposure status was significantly associated with Sgpt and creatinine (β = −16.3, 95% CI: 22.4–10.2; and β = 24.1, 95% CI: 16.7–31.5 respectively).

## 4. Discussion

The results of the present study showed significant differences in biological markers between the exposed and the reference populations. Residing in an area with high pesticide applications and reported incidences of pesticide poisoning cases suggests higher exposure compared to those residing in an area with no pesticide use and no history of reported cases of pesticide poisoning. In the present study, the measures of WBC, lymphocyte, platelet counts, MCHC, sgot and creatinine were higher in the exposed group compared to the reference group, whereas MCV, hematocrit, hemoglobin, and sgpt were lower in the exposed group compared to the reference group. Surprisingly, the AChE and BChE activities were higher in the exposed group compared to the reference group.

The decrease in hematocrit and MCV observed in the exposed group compared to the reference group might be explained by dehydration [36]. However, because erythrocyte deformability has been found when blood samples from healthy persons were exposed to acetylcholine in vitro, it is likely that chemicals such as pesticides with the potential to affect acetylcholine signaling may induce changes in erythrocyte size/volume [37]. Araoud et al. [32] in Tunisia and Azmi et al. [38] in Pakistan similarly found reduced MCV and hematocrit, as well as increased MCHC, WBC, lymphocyte, and platelet counts among pesticide-exposed individuals compared to controls. 

The high mean platelet count observed in the exposed group compared to the reference group may be related to a primary bone marrow disorder and release of too many platelets into the blood [43]. Previous studies have observed both increased and reduced platelet turnover in both acutely and chronically pesticide-exposed individuals [32,33,43,44,45]. These differences across studies may result from exposure to different pesticides or mixtures of pesticides, exposure dose, and exposure frequency and time [34,46,47,48]. However, high platelet counts may also be secondary to other causes such as allergic reactions, cancer, diabetes, and infections [49,50]. The etiology behind the elevated platelet counts observed in the previous studies has not yet been established. 

After adjusting for age and years of living in the study area, we observed that serum creatinine levels were significantly higher in the exposed subjects compared to reference subjects. Toe et al. [51] in Burkina Faso similarly showed high prevalence of liver and kidney dysfunction and significant associations between biochemical changes and kidney and liver function alterations in frequent users of insecticides. In addition, a study conducted in Pakistan [38] showed hematological and biochemical alterations combined with liver and kidney disorders in individuals exposed for over a month to multiple pesticides including cypermethrin, deltamethrin, polytrin, and diazninon. 

In the present study, after adjusting for age and years of living in the study area, sgpt was significantly lower in the exposed subjects compared to reference subjects, and the mean value was close to the lower normal range. Low sgpt may be associated with disease conditions such as fatty liver (early), liver congestion protein deficiency, and kidney failure. Increased sgpt level is a common finding in individuals exposed to relatively high doses of pesticides [52]. However, experimental studies in rats have demonstrated that pesticide (carbendazim) exposure induces a hormesis-type u-shaped dose response with reduced sgpt at low doses and increased sgpt at higher doses compared to controls. The changes in biochemical parameters were accompanied with histological lesions such as portal vein congestion, mononuclear cell infiltration, and hydropic degeneration of liver tissue in the test animals [53]. Reduced levels of sgpt were also found in rats exposed to diquat dibromide for four weeks [54], suggesting that various pesticides may affect sgpt differently depending on exposure conditions like dose and time.

However, as opposed to high levels, low creatinine and cholesterol levels are suggested to be of low physiological significance [55]. For all parameters—including monocyte count and sgpt level—the individual values were all close to the upper or lower reference indicating that none of references had extreme low or high values. 

The significantly higher esterase activity in sera of the exposed subjects compared to the reference subjects observed in the present study was not anticipated because studies analyzing esterase activity following exposure to pesticides mainly show reduced activity [56]. However, an opposite response may be produced following long-term as opposed to acute exposure [57]. The higher esterase activity in the exposed subjects may be explained by individual tolerance due to compensatory mechanisms [58,59]. Tolerance may develop in response to long-term exposure by increasing the number of receptors and/or enzymes and/or by increasing the metabolism and excretion of chemicals [60]. Increased esterase activity following long-term exposure to different AChE inhibitors has been previously seen in rats [61,62]. Goldfish chronically exposed to sub-lethal concentrations of OPs and carbamates showed an initial significant reduction in AChE activity in the brain followed by a gradual increase over time, suggesting that adaptive mechanisms are activated under long-term exposure conditions [63]. Studies in frogs and bees support these findings. For example, among frogs, exposure to sub-lethal doses of a mixture of pesticides induced cross tolerance to several pesticides [64]. Likewise, increased esterase activity was also demonstrated in bees exposed to low doses of neonicotinoids (AChE inhibitors) [65]. A plausible mechanism for the increased esterase activity in bees, and potentially also in mammals, was demonstrated by Williamson et al. [66], who found upregulation of AChE in the brain and gut tissues of bees chronically exposed to low doses of AChE inhibiting pesticides (coumaphos, aldicarb, chlorpyrifos, and donepezil). The opposite effect on esterase activity following long-term versus short-term exposure reported in the present and previous studies may have implications for the interpretation of AChE when used as biomarker for AChE-inhibiting pesticides such as carbamates, OPs and neonicotinoids. Additionally, AChE activity in plasma recovers slowly with time after acute exposure. For example, workers who were exposed to dichlorvos showed substantial plasma cholinesterase activity inhibition, immediately after exposure, however, with time activity showed an exponential pattern of recovery with a half-life of around 12 days, and complete recovery after 50 days [67].

The AChE inhibition test is a biomarker for exposure to AChE-inhibiting pesticides, but not for other groups of pesticides, including dithiocarbamates such as Mancozeb, which are among the most used pesticides in the study area. To detect the effects of exposure to multiple pesticides that may affect various tissues, organs and body systems, new biomarkers should be identified. Pesticides such as Mancozeb and Endosulfan, which are commonly used in mixture with OPs in the study area, may potentially add to the adverse effects among chronically exposed individuals. 

Several limitations of the present study should be noted. Because of limited resources, we had to restrict the number of participants to 99, which may have reduced the statistical power and consequently lead to lack of external validity. In addition, the use of a cross-sectional design makes it difficult to ascertain a causal link. Furthermore, while bio-monitoring for specific pesticides is a more sensitive method for measuring exposure, this could not be used in the present study because of resource constraints as well as rapid metabolism and excretion from the body. 

The methods used to assess exposure were not optimal to identify the individual exposure levels. However, in addition to the information given by the study participants, the significant difference in pesticide use between the two districts is well documented in scientific reports [11,39] and from pesticide dealers in the districts. Furthermore, the selection of participants was based on clear inclusion criteria defining exposure status and levels. This study is unique because it is reporting on a vulnerable but not institutionally protected group of female farm workers that has been scantly studied. Conducting a study in a hospital setting where privacy, care, and confidentiality are assured is thought to improve reliability and validity of the findings. Furthermore, regression methods were applied for analysis to control for potential confounders [68,69]. Despite the discussed limitations, the present findings are important for establishing risk of long-term exposure to mixtures of pesticides and establishing a relevant hypothesis for further investigation.

## 5. Conclusions

The results of the present study showed significant biological, biochemical, and esterase activity differences between the exposed and reference groups, suggesting higher risk of adverse health effects in the pesticide-exposed individuals. Identification of relevant biomarkers of exposure and effect in individuals chronically exposed to pesticides is important for diagnostic and preventive measures. Studies in vulnerable populations such as children and pregnant women exposed to harmful chemicals like pesticides are important. Further studies should assess the prevalence of pesticide-induced diseases in the exposed vulnerable study population. 

## Figures and Tables

**Table 1:** Selected social and physical characteristics of study subjects in the exposed and reference groups, given as n, mean and standard deviation (SD).

Variable	Exposed			Reference			*p*-Value
	*n*	Mean	SD	*n*	Mean	SD	
Age (years)	67	31.2	±6.2	30	27.8	±6.3	<0.02
Height (cm)	65	161.2	±7.5	27	162.5	±7.2	0.43
Weight (kg)	66	59.0	±9.6	26	58.3	±7.2	0.69
Years in current occupation	52	12.2	±6.4	10	14.3	±5.0	0.26
Years of living in the study area	63	16.8	±8.8	30	23.1	±9.2	0.00

**Table 2:** Pesticides commonly used in the study area (Arusha region, Tanzania) and the long-term neurological and hepato-nephrotoxic adverse effects reported in animal and/or human studies.

Active Ingredients (WHO Classification)	Common Names in the Area	Reported Effects of Repeated Exposure
OPs: Profenofos (II)	Selecron, Profecron, Mocron, Supercron,	Neurologic disorders
-	Tanzacron, Polytrin, Profit, etc.	Inhibits cholinesterase activity in high dose (animals)
OPs: Chlorpyrifos (II)	Dursban, Bamifos, Twigaphos, Duduba, * etc.	Persisting neurologic damage (humans); changes in organ weights (e.g., liver), cholinesterase enzyme inhibition, developmental neurotoxicity (DNA, synaptic, mitotic inhibition, etc.) (animals)
CMs: Carbosulfan (II)	Marshal, etc.	Cholinesterase enzyme inhibition, liver pathology
Pyrethroids (II): Permethrin, lambda-cyhalothrin, deltamethrine	Ninja, Karate, Ngao, Duduba, * etc.	Polyneuropathy and degeneration of ovary (rats), sperm abnormality
OC: Endosulfan (II)	Thionex, Thiodan, etc.	Neurological disorders, kidney and liver, endocrine disruption
Mancozeb (Unclassified)	Farmzeb, Oshothane, Milthane, Ivory, Dithane, Mancozeb *, Linkmil, * etc.	by EPA, reproductive effects (birth defects) and developmental effects due to metabolite ethylenethiourea; Hepatoxic and nephrotoxic (rats)

* Symbol shows formulation in combination. WHO classification II means moderately hazardous. OPs, Organophosphorus pesticides; CMs, Carbamates; OCs, Organochlorine pesticides.

**Table 3:** Hematological and biochemical comparisons between the exposed and reference groups. Results are given as mean and standard deviation (SD).

Parameter	Reference Values	Exposed Group (*n* = 69)	Reference Group (*n* = 30)	*p*-Value
White blood cell count (*0^9^/L)	3.1–7.8	6.8 ± 2.3	5.3 ± 1.2	<0.01
Lymphocyte count (*10^9^/L)	0.9–3.75	2.7 ± 0.8	2.2 ± 0.4	<0.01
Monocyte count (*10^9^/L)	0.15–0.39	0.6 ± 0.3	0.4 ± 0.6	0.05
Red blood cell count (*10^12^/L)	4.07–5.13	4.3 ± 0.5	4.3 ± 0.6	0.89
Mean corpuscular volume (MCV(fL))	77–94	74.7 ± 9.1	84.7 ± 7.6	<0.01
Haematocrit (%)	36–45	32.0 ± 4.6	36.2 ± 4.8	<0.01
Mean corpuscular haemoglobin (pg)	27.5–33.3	28.1 ± 5.4	28.4 ± 3.1	0.76
Mean corpuscular haemoglobin concentration, MCHC (g/dL)	33.4–37	37.4 ± 3.8	33.4 ± 1.2	<0.01
Haemoglobin (g/dL)	12.4–15.5	11.9 ± 1.9	12.1 ± 1.8	0.64
Platelet cell count (*10^9^/L)	150–400	374.1 ± 95.3	259.6 ± 77.4	<0.01
Sgpt (U/L)	up to 32	12.8 ± 6.1	29.5 ± 9.9	<0.01
Sgot (U/L)	up to 31	20.7 ± 8.9	17.6 ± 4.5	0.08
Cholesterol (mmol/L)	<5.2	4.1 ± 0.5	4.0 ± 1.2	0.83
Serum creatinine (μmol/L)	53.0–97.2	83.9 ± 6.6	61.8 ± 24.0	<0.01

Normal range values for blacks Bain et al. [41] 1984; Tikly et al. [42] 1987. Sgpt = up to 32 U/L, Sgot = up to 31 U/L, creatinine = 53.0–97.2 μmol/L, cholesterol ≤ 5.2 mmol/L. * multiplication symbol.

**Table 4:** Esterase enzyme activity and biochemical results comparison between the exposed and reference groups.

Esterase Name	Reference Values	Exposed (*n* = 68)	Reference (*n* = 30)	*p*-Value
AChE (mM/min/mg protein)	-	0.61 ± 0.24	0.17 ± 0.10	<0.01
BChE (mM/min/mg protein)	-	0.96 ± 0.39	0.23 ± 0.08	<0.01
Total protein concentration (g/dL)	6.0–8.3	7.4 ± 1.2	8.0 ± 1.0	0.01
